# Short message service (SMS) interventions for the prevention and treatment of sexually transmitted infections: a systematic review protocol

**DOI:** 10.1186/2046-4053-3-7

**Published:** 2014-01-16

**Authors:** Carole Lunny, Darlene Taylor, Jasmina Memetovic, Orion Wärje, Richard Lester, Tom Wong, Kendall Ho, Mark Gilbert, Gina Ogilvie

**Affiliations:** 1 BC Centre for Disease Control, 655 West 12th Avenue, Vancouver, British Columbia V5Z 4R4, Canada; 2 University of British Columbia, 2329 W Mall, Vancouver, British Columbia V6T 1Z4, Canada; 3 Public Health Agency of Canada, 130 Colonnade Road, AL 6501H, Ottawa, Ontario K1A 0 K9, Canada; 4 Department of Emergency Medicine, UBC Faculty of Medicine, Room 3300, 910 West 10th Avenue, Vancouver, British Columbia V5Z 1 M9, Canada; 5 eHealth Strategy Office, UBC Faculty of Medicine, Room 215, 855 West 10th Avenue, Vancouver, British Columbia V5Z 1 L7, Canada

**Keywords:** short message service, cell phones, mobile health, HIV, chlamydia, gonorrhea, syphilis, hpapillomavirus, herpes simplex virus, Sexually transmitted infections

## Abstract

**Background:**

Globally, the incidence of sexually transmitted infections (STI) is rising, posing a challenge to its control and appropriate management. Text messaging has become the most common mode of communication among almost six billion mobile phone users worldwide. Text messaging can be used to remind patients about clinic appointments, to notify patients that it is time for STI re-testing, and to facilitate patient communication with their health professionals with any questions and concerns they may have about their sexual health. While there are a handful of systematic reviews published on short message service (SMS) interventions in a variety of health settings and issues, none are related to sexual health. We plan to conduct a systematic review to examine the impact text messaging might have on interventions for the prevention and care of patients with STIs.

**Methods/Design:**

Eligible studies will include both quantitative and qualitative studies published after 1995 that discuss the efficacy and effectiveness of SMS interventions for STI prevention and management using text messaging. Data will be abstracted independently by two reviewers using a standardized pre-tested data abstraction form. Inter-rater reliability scores will be obtained to ensure consistency in the inclusion and data extraction of studies. Heterogeneity will be assessed using the I^2^ test and subgroup analyses. A nonhypothesis driven inductive reasoning approach as well as a coding framework will be applied to analyze qualitative studies. A meta-analysis may be conducted if sufficient quantitative studies are found using similar outcomes.

**Discussion:**

For this protocol, we identified ten related systematic reviews. The reviews were limited to a particular disease or setting, were not exclusive to SMS interventions, or were out of date. This systematic review will be the first comprehensive examination of studies that discuss the effectiveness of SMS on multiple outcomes that relate to STI prevention and management, covering diverse settings and populations. Findings of the systematic review and any additional meta-analyses will be published and presented to our key knowledge users. This information will provide the evidence that is required to appropriately adopt text messaging into standard practice in STI care.

## Background

Increasing rates of reported sexually transmitted infections (STIs) remain a major public health challenge worldwide. Despite active and passive surveillance activities and multiple interventions aimed at increasing case finding and treatment, human immunodeficiency virus (HIV), chlamydia (CT), gonorrhea (GC), syphilis, herpes, and human papillomavirus (HPV) infections impose a large burden on health resources [1,2].

In the past two decades, the population use of new technologies such as mobile phones and the internet has exploded. The Canadian Wireless Telecommunications Association figures showed that almost 27 million Canadians, representing more than 81% of the Canadian population, subscribed to mobile phones in 2012 [3]. Of those who own smart phones in Canada, according to Rogers Communications survey in 2012, text messaging was noted to be the top application (88% users) [4]. Furthermore, a Statistics Canada 2010 survey revealed that 78% of Canadian have a cell phone, and wireless-only homes increased to 13% from 8% in 2008 [5].

Text messaging is commonly used in a variety of medical contexts. Text messaging allows patients and providers to ‘interact’ via two-way communication [6], which can allow for enhanced support by health-care providers to confirm medication taking [7,8], to enable patients to ask medication questions to pharmacists [9], and to alert clinic staff of problems [10]. SMS messages can be customized to fit the needs of specific individuals by delivering tailored messages that are more likely to catch the individual’s attention and be perceived as personally relevant and interesting [11]. Moreover, because messages exchanged between health-care providers and clients are stored on the device, there is the potential for them to become part of the client’s health-care record.

SMS has been used in other health contexts in the form of smoking cessation [12-14], cancer [15], diabetes [16-23], asthma [24-34], diet or weight management [35], obesity [36], and reminder programs [37-43]. In the context of sexual health services, SMS has been used in the form of appointment reminders [44,45], STI rescreening reminders [46,47], provision of STI results [48-51], communication of STI information [10,52], sexual health promotion [53,54], and assistance with contact-tracing [55-58]. It has also been shown to decrease the amount of time from diagnosis to treatment among positive chlamydia patients [49], increase the rate of retesting among high risk groups [59], and reduce the amount of missed clinic appointments [10,44]. Others report that sexual health knowledge and behavior is increased by delivering educational messages via text message [53,54]. Because younger people have higher risk of acquiring STIs such as GC and CT, text messaging, which is commonly used by this segment of the population, can be an effective tool to reach and help them in STI management and care. Today, text messaging and email managed on handheld devices are overtaking traditional voice calling for personal communication in Europe and North America. However, despite widespread use, there is little consensus about the actual impact that SMS interventions have on the prevention and control of STIs. The purpose of this systematic review is to examine the use of SMS to improve the treatment and prevention of STIs. Our research will attempt to answer the following questions: (a) What are the various ways that SMS use has been proposed to improve STI prevention and management in quantitative studies with control groups? (b) What are the potential benefits and harms for participants of SMS interventions related to STI programs? (c) What are the experiences and perceptions of people involved in STI-related SMS interventions? and (d) Why does an intervention work (or not), for whom, and in what circumstances?

## Methods/Design

### Inclusion/exclusion

The following inclusion/exclusion criteria are based on Patient and Problem, Intervention, Comparison and Outcome (PICO) domains.

#### ***Study design***

Eligible studies will include randomized and non-randomized controlled trials, pre- and post-test designs, non-experiment observational (cross-sectional, case-series, case studies) and qualitative papers that examine the benefits and other impacts of SMS interventions on STIs.

#### ***Population***

Individuals of any age who use cell phones and have been involved in an intervention that uses text messaging as patient support to improve the treatment and prevention of STIs will be included.

#### ***Intervention***

SMS or text messaging interventions that are delivered through a mobile electronic device to improve the treatment and prevention of STIs will be included.

#### ***Comparator***

The comparison is the usual standard of care, or in the case of a randomized control trial, the comparison is the control condition.

#### ***Outcome***

The *a priori* primary outcomes of interest include: (a) clinical outcomes (HIV viral suppression, STI clearance, STI re-infections); (b) adherence (for example, percentage of missed appointments, adherence to medication), (b) STI testing (for example, rates of primary testing, re-testing), (c) changes in STI knowledge or risk behavior (for example, increased condom use), (d) uptake of SMS for partner notification, (e) acceptability of SMS for STI interventions, and (f) any cost-effectiveness assessments. Secondary outcomes are: notification of test results, condom use, mental health outcomes (for example, anxiety and depression scores), quality of communication with clinicians, quality of care, feasibility of program delivery and privacy impact of SMS messages (for example, content of messages). Outcomes that are similar will be grouped for quantitative synthesis. Outcomes will be grouped based on an objective or subjective class (Table 1). We will include other outcomes of interest identified during the literature review.

**Table 1:** Outcomes classified on objective or subjective criteria

**Objective/semi-objective**	**Subjective**
● Mortality	● Mental health outcomes
● Suppression of HIV viral load	● Quality of life/functioning
● Sexually Transmitted Infection (STI) clearance	● Satisfaction with/Quality of care
● STI re-infection	● Quality of communication
● Withdrawals/drop-outs	● General physical health
● Time to testing for an STI or HIV	● Adverse events
● Time of symptom onset to seeking medical treatment (first time, recurrence)	● Continuation of condition
● Time to uptake of diagnosis or treatment	● Cost-effectiveness
● STI testing rates	● STI knowledge and behaviour
● Correct clinical diagnosis or assessment	● Communication uptake regarding STIs
● Improvement in condition (i.e. signs and symptoms)	● Feasibility of program delivery
● Number and proportion of partners notified by short message service (SMS)	● Privacy impact/assessment
● Cost savings/reduction	● Notification of test results
	● Condom use

#### ***Exclusion criteria***

The following data will be excluded: commentary or opinion publications without new data, publications before 1996, research that does not include use of SMS/text messaging, research that uses PDAs other than PDA phones, and studies with an email/social network-based/landline telephone intervention. Studies using PDAs only (and not PDA phones) will be excluded because of their relatively uncommon use with most populations during their peak popularity (for example, often used by physicians but not their patients).

### Search strategy

The databases that will be searched for journal articles, reports, editorials and abstracts include Ovid (for example, Cochrane Database of Systematic Reviews, Medline, Embase), Web of Knowledge (for example, Biosis, Web of Science), and EBSCO (for example, PsycINFO, ERIC, CINHAL, *etcetera*). The gray literature will be searched for reports, dissertations, conference proceedings and mobile health-related websites. Our search will include English and non-English-based databases. Since SMS is relatively new, the search will be limited to articles published between 1996 to August 2013. The following STI journals will be hand-searched by an inhouse librarian: *Sexually Transmitted Diseases*, *Sexually Transmitted Infections*, and *AIDS Patient Care and STDs*.

### Search terms

Medical Subject Headings (MeSH), subject headings and keywords will be created by using language that describes text message interventions for STIs. Search terms will include but will not be limited to: mobile health, mHealth, cell phone, mobile phone short message service, SMS, MMS, communication technologies, patient monitoring devices, wireless technologies, STI testing, sexually transmitted diseases, sexually transmitted infections, HIV, chlamydia, gonorrhea, herpes, *Trichomonas vaginalis*, and syphilis. Boolean combinations will create more specific searches using Ovid MeSH terms as the standard for developing a search strategy for each database.

A health librarian will be consulted to ensure the optimal search strategy is being conducted. In addition, backward and forward citation searches of included studies, relevant evidence reviews and reports will also be done. Email letters will be sent out to scholars in leadership and other related fields to ask them to review the list of studies that we included and to suggest other studies that they thought might be missed. This list will be expanded upon during the data collection phase.

### Study selection and extraction process

One reviewer will be responsible for creating a search strategy and will store all identified references in a shared RefWorks account. Once duplicates are manually removed, all publications found will be exported into an MS Access database. Two reviewers will then independently read the titles and abstracts of the identified articles and determine eligibility based on the specified inclusion/exclusion criteria. Any disagreements between the reviewers will be resolved by a third reviewer. Once the subset of publications meeting inclusion criteria is finalized, each publication will be reviewed and its characteristics documented using a standardized pre-tested data extraction form. These forms will capture: the purpose of the SMS intervention, duration of the intervention, delivery frequency of text messages, study design, setting and outcomes. The reviewers will attempt to contact the authors of studies that are missing key data. The reviewers (CL, JM, and OW) will translate included studies written in French, Spanish. German, Mandarin or Korean, or use online translation software.

Two reviewers will assess the studies with disagreement resolved by a third reviewer, and inter-rater reliability will be measured using kappa statistics. An inter-rater Kappa score will be assessed during the inclusion/exclusion phase of review, to ensure that a Kappa score at or above 0.8 is reached as measured by Cohen’s Kappa (k) statistical test [60]. If the measure falls below our threshold for high correspondence (0.8), the three reviewers will discuss until agreement is reached.

### Methodological quality

The methodological quality will be assessed using appropriate tools, including the Cochrane Collaboration’s Risk of Bias tool for randomized controlled trials, the Cochrane Effective Practice and Organization of Care group’s tool for quasi-experimental designs, and the risk of bias tool developed in Waddington *et al*. [61] study for regression-based studies (with special attention to confounding) [61]. Other observational studies will be assessed using the NOS score (Newcastle-Ottawa Quality Assessment Scale) [62]. The NOS score rates quality based on high risk (1 to 3 stars), medium risk (4 to 5 stars), or low risk (6 to 9 stars) NOS score [62]. If data allows, we will rate the overall quality of body of evidence using the GRADE system as it incorporates ratings for consistency, directness, and precision per outcome across multiple studies in addition rating the overall validity and risk of bias (http://www.gradeworkinggroup.org/).

It is usually necessary to consider the reliability or validity of the actual outcome measure being used (for example, several different scales can be used to measure quality of life or psychological outcomes). The reviewers will meet to discuss any differences in the interpretation of the scales measuring semi-objective and subjective outcomes. For bodies of evidence that include observational research, we will also systematically assess the characteristics of each outcome, including dose–response association, plausible confounding that would change the observed effect, and the strength of association. We have registered our protocol with the Preferred Reporting Items for Systematic Reviews and Meta-Analyses (PROSPERO registration number CRD42013006503).

#### ***Data analysis***

##### 

**Qualitative synthesis of studies** We will describe the clinical and methodological characteristics of the included studies, including their size, inclusion or exclusion of important subgroups, timeliness, and other relevant factors, both qualitatively and by using tables of study characteristics [63]. The strengths and limitations of individual studies and patterns across studies will be assessed and we will explain how design weaknesses or execution of the study (or groups of studies) could bias the results.

##### 

**Quantitative analysis** If the systematic review includes randomized controlled trials or observational studies, we will conduct meta-analysis and the Cochrane Collaboration’s Review Manager 5.0 will be used [64]. In the first analysis, a fixed-effects model will be used, and a random-effects model will check against it to ascertain its robustness. We will extract comparable effect size estimates from included studies, together with 95 percent confidence intervals. Where possible, we will calculate standardized mean differences (SMDs) for continuous outcome variables, and risk ratios (RRs) for dichotomous outcome variables.

Treatment effects will be calculated as the ratio of, or difference between, treated and control observations in a consistent way, such that outcome measures are comparable across studies. Thus, an SMD greater than zero (RR greater than 1) will indicate an increase in the outcome under the intervention as compared to the comparison. An SMD less than zero (RR between 0 and 1) will indicate a reduction under the intervention as compared to the comparison. An SMD equal to (or insignificantly different from) zero (RR equal to 1) will indicate no change in outcome over the comparison. Whether these relative changes represent positive or negative impacts will depend on meaning of the outcome in the context of the program being evaluated. We will only include one effect estimate per study. Where studies report multiple effect sizes according to subgroups of participants, we will report data on subgroups separately.

If statistical heterogeneity is observed, a random-effects model will be used. Statistical heterogeneity between studies will be examined visually using a I^2^ statistic and a chi-squared test (a chi-squared *P* value of less than 0.10 or an I-squared (I^2^) value equal to or more than 50% will be considered indicative of heterogeneity [65]. Furthermore, if heterogeneity is detected, subgroup analyses and meta-regression will be performed to identify factors that explain the heterogeneity. The factors we identify *a priori* are: (a) type of study design, (b) type of intervention, (c) purpose of the SMS intervention, (d) duration of intervention, (e) study setting, (f) sex ratio, (g) age groups (for example, adolescents, young adults, older adults), (h) quality rating, (i) type of outcome (for example, STI type, type of device used (Apple android, *etcetera*), number of reminders, number of appointments missed, contraceptive used), (j) English versus non-English literature, and (k) published versus unpublished literature. To evaluate the possibility of publication bias, we will use the Peters test and a color-enhanced funnel plot that will be done using STATA software (StataCorp. 2011. *Stata Statistical Software: Release 12*. College Station, TX: StataCorp LP.) [66,67].

##### 

**Qualitative analysis** We will employ interpretive description to answer the qualitative questions in our review [68]. Interpretive description, developed by Thorne *et al*. in 1997, is applied to qualitative research findings to solve a clinical problem as opposed to exploring a topic as an end goal (Thorne [68]). This strategy has two phases: (a) deriving findings inductively from data without imposing predetermined hypotheses, and (b) generating results that apply to a real-world clinical practice. We will review qualitative data from included studies, develop a coding framework to code data using NVivo, and hold weekly discussions to resolve conflicts and arrive at final conclusions.

## Discussion

While there has been an explosion in the number of articles and studies on text messaging use in health interventions, few reviews have conglomerated the literature related directly to SMS and STIs. Ten systematic reviews about mobile interventions for promoting sexual health were identified ([69-78]; Additional file 1). The reviews were limited to a particular disease or setting, were not exclusive to SMS interventions, or were out of date.

SMS technology and use has evolved and has expanded in multiple health-care settings, and the number of studies related to SMS interventions for STI has dramatically increased since previous reviews were done. Although the reviews by Horvath *et al.*[69], Chavez *et al.*, Zou *et al.* and Velthoven *et al.*[70-72] present important findings with respect to text messaging and sexual health outcomes, they do not cover multiple efficacy and effectiveness outcomes of SMS interventions for a broad spectrum of STI prevention and control. Broad reviews such as the one by Sørensen *et al.*[73] and by Chavez *et al.*[70] on the impacts of eHealth and other digital media may be useful; however, the girth of information on non-SMS information and communication technologies often overshadows the focused questions that health providers may seek on mHealth specific interventions. There have been a number of recent research papers specific to SMS and STIs with higher quality evidence. Therefore, an up-to-date review with a synthesis of current evidence is warranted.

### Significance of this review

A host of new remote monitoring and communication technologies are available, allowing providers to interact with patients anywhere and anytime, and patient engagement is key to managing STIs [79-81]. Cell phones, personal devices that are highly convenient to use, are particularly suited for leveraging the time and expertise of providers in communication with their patients. This prompt and personal communication can effectively engage patients in their knowledge acquisition and motivate them towards effective self-care. Like any consumers, patients as the primary users of health services prefer to have many options for communicating with their providers. Text messaging via mobile phones could not only provide a convenient option [82], but could also decrease the need for booking repeat appointments through timely doctor-patient communication, thereby reserving scarce health resources for those who need face-to-face encounters with clinicians.

This synthesis is particularly important as there is a lack of quality evaluations of SMS interventions on multiple outcomes affecting STI management. Researchers have attempted to empirically assess the effectiveness of SMS interventions but the data are sparse and have been collected with small sample sizes. Furthermore, many systematic reviews attempting to assess SMS interventions were conducted before randomized controlled trials on key outcomes were published. Synthesizing data from numerous studies will provide greater confidence in the effectiveness of these interventions, especially if the data allows for a meta-analysis. Moreover, there are conflicting results about the acceptability of some SMS interventions such as receiving results of laboratory tests as well as interventions for partner notification. A knowledge synthesis will provide more power to assess these conflicting reports and provide a pooled estimate of acceptability with reduced uncertainty.

Text messaging shows immense potential for prevention and management of STIs. However, there is a lack of consensus on its acceptability, feasibility and cost-effectiveness for different STIs, populations, settings and uses. The systematic review will be the first evaluation of the scope of SMS use in clinical and community settings for all levels of STI prevention and treatment. This information will provide the evidence that is required to make text messaging standard practice in STI care.

## Abbreviations

CT: Chlamydia; GC: Gonorrhea; HPV: Human papillomavirus; NOS: Newcastle-Ottawa quality assessment scale; PICO: Patient and problem, intervention, comparison and outcome; PROSPERO: Preferred reporting items for systematic reviews and meta-analyses; RR: Risk ratio; SMD: Standardized mean difference; SMS: Short message service; STI: Sexually transmitted infection.

## Competing interests

The authors declare that they have no competing interests.

## Authors’ contributions

CL drafted, edited and finalized the manuscript. DT, JM and OW edited the manuscript. JM and OW did the literature searching. GO, RL, and MG contributed to the research design. DT, GO, MG, KH, MK, TW participated in writing the grant application. All authors read and approved the final manuscript.

## Supplementary Material

Additional file 1 **Summary of ten systematic reviews found through a scoping review **[83]**.** Click here for file
