# Supplementary material for: Short message service (SMS) interventions for the prevention and treatment of sexually transmitted infections: a systematic review protocol
Source: Syst Rev. 2014 Jan 16;3:7. doi: 10.1186/2046-4053-3-7 (PMC3904420; doi:10.1186/2046-4053-3-7)
Supplement: Additional file 1 — Summary of ten systematic reviews found through a scoping review [83]. [file 2046-4053-3-7-S1.docx]

Additional file 1: Appendix A: Summary of ten systematic reviews found through a scoping review

For our protocol, a scoping review was conducted of OvidSP, EBSCO and Web of Knowledge databases using the words mHealth and text messaging combined with sexually transmitted infections, with ‘systematic review’ in the title. The following ten systematic reviews were found:

Lim *et al*. (2008) [74]

Lim et al. in 2008 conducted a systematic review of the uses of mobile phone text messaging for sexual health [74]. This is a comprehensive review of manuscripts that were published up to 2007 that used SMS in sexual health with a descriptive summary of SMS interventions used for clinical management, sexual health services, and health promotion from nine manuscripts (five cross-sectional studies, one randomized controlled trial, one descriptive study and two papers of unknown study design). Although this review is the first known systematic evaluation of studies on SMS and sexual health, it lacks the landmark randomized trials published after 2008 on the efficacy of SMS interventions on adherence and clinical endpoints of STIs.

Sørensen et al. (2008) [73]

Sørensen et al. conducted a systematic review of ‘information and communication technology’ (ICT) systems (including both telemedicine and ehealth) for antiretroviral treatment management among individuals with HIV/AIDS in South Africa [73]. Once again, this study was not focused on SMS interventions exclusively and was restricted to findings from South Africa.

Déglise *et al*. (2012) [75]

Déglise et al. conducted a systematic review of SMS interventions on communicable and noncommunicable disease control in developing countries [75]. Out of 98 studies, they identified 29 HIV- and 7 sexual health-related studies that investigated self-management, compliance and acceptance of SMS. Although this was the first review to consider acceptance of SMS, it was not exclusive to sexual health and only included studies in low- to middle-income countries.

Horvath *et al*. (2012) [69]

This review was published through the Cochrane Collaboration and sought to determine whether mobile phone text-messaging is efficacious in enhancing adherence to antiretroviral therapy in patients with HIV infection [69]. Despite searching the literature from Jan 1980 to Nov 2011, only two articles were included in the synthesis: one trial comparing SMS against standard care [10], and one comparing short daily, long daily, short weekly, and long weekly messages against standard care [83]. This review is specific only to HIV treatment adherence, whereas we will synthesize the results of SMS interventions for a broad range of STIs.

Zou *et al*. (2012) [71]

This review consisted of eight studies that evaluated a broad array of interventions, including SMS, aimed at increasing screening for bacterial sexually transmitted infections among men who have sex with men (MSM) [71]. Although this review provides important information about SMS interventions, it is not focused primarily on SMS interventions and therefore does not offer the synthesis we aim to provide.

Braun *et al*. (2013) [76]

Community health workers are increasingly relying on mobile technologies to facilitate ongoing care of their clients, especially in low-resource settings [76]. Braun et al. conducted a systematic review on the use and acceptability of mobile health by community health workers for health education, promoting adherence and testing. It includes 25 full-text studies on maternal and child health, HIV/AIDS, and sexual and reproductive health. This review offers a unique perspective of acceptability of mobile phones to health workers, which will be important for program implementation. Although most of the studies included in this review focus on STI prevention, it was not specifically focused on text messaging interventions for sexual health.

Butler *et al*. (2013) [77]

Similar to the Cochrane review published in 2012 by Horvath et al., this systematic review included publications that discussed multiple mHealth interventions (including voice messaging) for improving ART adherence [77]. Out of the 453 identified references up to 2012, eight focused on text messaging. {AU Query: Please define ‘ART’ upon first use and add to list of abbreviations used.}

Chavez *et al*. (2013) [70]

Chavez et al. conducted a systematic review looking at all digital media technology (including the internet and interactive videos) on the primary prevention of sexually transmitted infections in multiple settings [70]. The 26 studies focused on STI knowledge, intent to use condoms and abstinence. This review was limited to the role of SMS interventions for primary prevention of STIs, and therefore did not include outcomes related to the other tiers of prevention (for example, STI testing and biomarkers). The current review will expand the search to include all levels of prevention and surveillance using SMS technology.

Gentry *et al*. (2013) [78]

This review presented research on mobile and landline-delivered interventions for HIV/AIDS prevention and management, finding close to 15,000 citations including 11 RCTs [78]. Primary outcomes included adherence, virologic endpoints, depressive symptoms and changes in risky sexual behavior. It includes a meta-analysis of three studies that did not yield positive results. We will implement a similar systematic review protocol as Gentry et al., with study inclusion criteria that include RCTs, quasi-randomized controlled trials, and cohort studies, but will exclude studies with landline phone as the primary intervention.

Velthoven *et al*. (2013) [72]

This review included 21 studies that were specific to SMS interventions for HIV/AIDS care, and provided summaries on adherence, communication with care providers, HIV prevention and testing [72]. However, the studies included did not investigate acceptability and STI knowledge, and were specific to HIV/AIDS.
